# Association between physical activity and physical health in German children and adolescents - results from the MoMo Longitudinal Study

**DOI:** 10.1186/s12889-025-21684-w

**Published:** 2025-02-13

**Authors:** Simon Kolb, Alexander Burchartz, Leon Klos, Darko Jekauc, Claudia Niessner, Alexander Woll

**Affiliations:** Institute of Sports and Sports Science, Karlsruhe Institute of Technology, Karlsruhe, Germany

**Keywords:** Cardiorespiratory fitness, Body composition, Recurrent headache, Motor performance, Strength

## Abstract

**Background:**

Adequate physical activity is essential to maintain and improve physical health, including in adolescents and young adults. A significant part of physical activity is sports activity. However, few longitudinal studies cover the transition from adolescence to early adulthood. Therefore, the aim is to assess how the maintenance or a change in sports activity during this transition relates to physical fitness, BMI, and recurrent pain.

**Methods:**

Data of 947 adolescents aged 11 - 17 years (53 % girls) from the Momo baseline (2003 - 2006) that were followed up at MoMo Wave 1 (2009 - 2012) were used. Sports activity was categorized (> 240 min/wk sports activity vs. $\le$ 240 min/wk) and change patterns over time were calculated (maintain active, maintain passive, increasing, decreasing). Separate ANOVAs were used to analyze the difference in cardiorespiratory fitness (CRF), push-ups, standing long jump, and BMI at wave 1 between the sports activity groups. A logistic regression was used to assess the difference in the occurrence of recurrent headaches at wave 1 between groups. All analyses were controlled for sex and age, and post-Hoc-Tests were conducted where applicable.

**Results:**

The analysis revealed significant differences between the activity groups at the follow-up for CRF (F(3, 502) = 13.65, *p* < .001), push-ups (F(3, 599) = 10.45, *p* < .001), and standing long jump (F(3, 601) = 12.03, *p* < .001). There were no significant differences in BMI between the groups (F(3, 610) = 0.08, *p* = .970). The odds for the low active group were two times higher to report recurrent headaches at the second measure compared to the consistently active ones.

**Conclusion:**

Participating in sports regularly from adolescence to early adulthood is associated with the strongest health benefits. Nevertheless, those who were less active initially but increased their sports activity had similar health outcomes, highlighting the importance of sports activity as part of a healthy lifestyle in young adulthood.

## Introduction

Adequate levels of physical activity (PA) are essential for improving or maintaining health [1, 2]. As the foundation for health and PA is laid in childhood and adolescence, it is crucial to examine this relationship early on.

As part of a systematic review, a group of 27 experts in physical activity research with children and adolescents identified 11 relevant indicators to assess health in this population. Seven of these were categorized as critical or primary and four others as important or secondary [1]. For this study, we looked at body composition, cardiorespiratory fitness (CRF), musculoskeletal health, and pain which are proxy measures for three critical indicators body composition, physical fitness, and harms.

Overweight and obesity are not only the leading risk factors in the adult population but are also a major concern for children and adolescents. Adolescent overweight and obesity are associated with an increased risk of chronic diseases, including type 2 diabetes later in life [3]. Although weight loss and maintenance can be challenging [4], preventing excessive weight gain during childhood and adolescence is crucial. Data from the International Children’s Accelerometer Database (ICAD) showed associations between moderate-to-vigorous physical activity (MVPA) and lower BMI and waist circumference (WC) z-scores that were particularly pronounced at higher percentiles, suggesting that promoting MVPA could reduce the number of children and adolescents in the upper tail of the BMI and WC distributions [5]. Given that overweight and obese children or adolescents often continue to struggle with weight into adulthood [6], early intervention is essential.

Physical fitness can be seen as a comprehensive assessment of numerous, if not all, bodily functions, including the musculoskeletal and cardiorespiratory systems, among others. These functions play an essential role in the execution of daily tasks, PA, or physical exercises. This makes PF itself an important health marker [7]. An aspect of PF is CRF, which was an important health resource for the prevention and management of non-communicable diseases such as cardiovascular disease [8], cancer [9], and diabetes mellitus [10]. It also contributed significantly to reducing the risk of premature mortality [9]. A recently published study of Icelandic children revealed that adolescence was a significant milestone in the development of CRF. The findings advocated early measures to improve CRF in later life [11]. Another aspect of PF is muscular fitness (MF), which refers to the ability to exert force against resistance. MF was influenced by muscle size, fiber activation, and group coordination, making it difficult to measure with a single test. Important health-related components included maximal, explosive, endurance, and isokinetic strength [7]. It played an important role in daily activities, exercise performance, and disease prevention [12].

Recurrent pain was not only a problem in adults; it also affected children and adolescents [13–16]. It can significantly disrupt daily activities, leading to increased school absences and reduced participation in sports or social events [15, 17]. In addition, recurrent pain affected well-being, which could lead to a reduced quality of life (HRQoL) and emotional challenges such as anxiety and depression [18, 19]. Although scientific evidence was limited, PA, especially aerobic exercises, was often recommended for migraine patients [20, 21].

Childhood and adolescence are pivotal stages in life, marked by significant physiological and psychological changes. During these stages of their life, people form lifestyle habits and behaviors, both healthy and unhealthy, that can shape their adult lives and overall health [22]. For example, the transition from secondary school to young adulthood is an important developmental phase in which several serious changes occur simultaneously and within a short period [23, 24]. Recent comprehensive studies have explored the relationship between adolescent physical activity and its immediate and long-term effects on health [7].

To be able to map the changes during such transitions, studies are needed that examine the same participants at different points in time. Unfortunately, these longitudinal analyses are precisely the studies whose importance is repeatedly emphasized and of which there are still too few [1, 2]. Those studies are costly and resource-intensive, especially on a large scale, which is one of the possible reasons for their scarcity.

The purpose of this article was to assess the influence of maintaining, increasing, or decreasing sports levels on different physical health indicators over 5 years in adolescents in transition to adulthood using data from a national longitudinal study from Germany.

## Methods

### Study design and participants

Data from the German Motorik-Modul Longitudinal Study (MoMo) were used for this analysis. MoMo [25–28] was initiated in 2003 as an in-depth module of the German Health Interview and Examination Survey for Children and Adolescents (KiGGS), which is part of the German Health Monitoring of the Robert Koch-Institute (RKI). Similar to KiGGS, MoMo combined a cross-sectional survey at each wave (T1: 2003–2006, T2: 2009–2012, T3: 2014–2017, T4: 2018–2022), and a longitudinal sample that is followed up since the baseline measurement [25–27]. At baseline, a total of 17,641 children and adolescents aged 0 to 17 years with primary residence in Germany participated in the representative KiGGS-Study. The study population was selected using a stratified multistage probability sample with three evaluation levels. In the first step, 167 municipalities were selected from the inventory of municipalities in Germany. Then a stratified sample of participants aged 0 to 17 years was randomly selected from the register of the municipalities. A detailed description of the sampling procedure is given by Kurth et al. [25]. Finally, a subsample of KiGGS was selected to participate in MoMo. The studies were approved by the Ethics Committee of Charité/Universitätsmedizin Berlin and were conducted according to the Declaration of Helsinki.

Between 2003 and 2006, 4,529 children and adolescents aged 4 to 17 years from all over Germany participated in the MoMo baseline. For the following analysis, only participants between 11 and 17 years old at baseline who also completed the measurement in Wave 1 (T2: 2009–2012) were selected (*n* = 947).

### Variables

#### Anthropometric measures

Standing height without shoes was assessed using a stadiometer (Seca, Hamburg, Germany) with an accuracy of 0.1 cm. Body mass in light clothing was determined using an electronic scale (Seca, Hamburg, Germany) with an accuracy of 0.1 kg. BMI was calculated by dividing body mass by the square of height.

#### Cardiorespiratory fitness

CRF was evaluated using the Physical Working Capacity 170 (PWC170) cycle ergometry test, performed on a stationary bicycle (Ergosana, Germany). The initial workload was calculated based on body mass and incrementally increased every 2 minutes. More information on the protocol can be found elsewhere [26]. Participants followed this progressive protocol until their heart rate exceeded 190 beats per minute (bpm) for at least 15 seconds, the pedaling rate dropped below 50 rpm for at least 20 seconds, or they decided to stop because of exhaustion. Heart rate was recorded immediately before each increase in workload. The power output (in watts) at a heart rate of 170 bpm (PWC170) was determined by interpolating or extrapolating the measured data in Excel.

#### Musculoskeletal fitness

The MoMo test battery assesses various aspects of physical fitness [29], including explosive strength of the lower extremities and muscle endurance of the upper extremities. To assess the explosive strength of the lower extremities, the subject performs a standing long jump task. The participants try to jump as far as possible from a standing position without losing their balance. Each subject has two attempts, and the furthest attempt is counted.

Muscle endurance of the upper extremity is measured using a standardized form of push-ups. In this test, the participant starts in the prone position, smoothly transitions into the push-up position, lifts one hand off the ground, touches the other hand, and returns to the starting position. The repetition ends by bringing the hands together behind the back while still in the prone position. The test leader demonstrates the correct execution. Participants have 40 seconds to complete as many correct repetitions as possible. One round was completed.

#### Self-reported pain

Participants self-reported pain experienced in the three months prior to the survey using a questionnaire. They were presented with a list of different pain locations and asked to indicate whether the pain had occurred once, recurred, or had not occurred during that period. The pain locations included the head, abdomen, back, ears, eyes, lower abdomen, arms/hands, legs/feet, throat, teeth, and thorax. The female participants were additionally asked about menstrual pain. In the current study only headache was included as a measure of physical pain because previous analyses had shown it to have the highest prevalence in the study population [19, 30, 31]. For the analysis, the responses were categorized into two groups: recurrent pain versus no pain or pain occurring only once.

#### Physical activity

Physical activity in different settings (sports clubs, leisure time, school, daily life) was assessed via a questionnaire. In terms of reliability and validity, the MoMo-PAQ is similar to other internationally published physical activity questionnaires for adolescents [32].

In this study, a sporting activity index is used as a measure of PA. The index sums up the total minutes of physical education, extracurricular sports activity in school, leisure time sports activity outside a sports club, and leisure time sports activity within a club sport per week.

### Data analysis

Data analysis focused on differences based on the activity level of the participants. Therefore, the sample was grouped based on the sport activity index at the first measurement. The group median was used as the cut-off value for classifying the groups, resulting in two groups of approximately equal size. Participants were categorized as either highly active if they exercised for more than 240 minutes per week, or as low active if their total activity was below this threshold.

For the analysis of changes in health-related activity behavior, the sporting activity index at T2 was classified in the same way and the activity categories of both measurements were combined. This resulted in four groups: the consistently high active, the consistently low active, as well as the two switcher groups, the increasers, and the decreasers. To analyze the difference in BMI, CRF, and musculoskeletal fitness at the second time point between the sports activity groups, we used separate ANOVAs for each outcome with age at baseline and sex included as covariates. Pairwise comparisons of means were tested using the Tukey method. For the effect of PA development on recurrent headaches, logistic regression with the PA group as a factor was used because the outcome variable is dichotomous. Here, the consistently active group was used as a reference, and Wald statistics was used to check for significant differences between the estimates of the sports activity group.

All analyses were performed in R [33] and the level of significance was set at .05 for all statistical analyses.

## Results

The sample consists of a total of 947 (53 % female) subjects that participated in baseline (T1: 2003–2006) and wave 1 (T2: 2009–2012). Sex, mean age at baseline, and total sport minutes at baseline stratified by PA group are presented in Table 1.

**Table 1:** Sample description stratified by activity group

	High active^b^ (*N* = 233)	Low active^c^ (*N* = 367)	Decreaser^d^ (*N* = 247)	Increaser^e^ (*N* = 100)	Overall (*N* = 947)
**Sex**					
Male	155 (66.5 %)	116 (31.6 %)	124 (50.2 %)	52 (52.0 %)	447 (47.2 %)
Female	78 (33.5 %)	251 (68.4 %)	123 (49.8 %)	48 (48.0 %)	500 (52.8 %)
**Age at baseline (in years)**					
Mean (SD)	13.8 (1.94)	14.6 (1.97)	14.1 (1.94)	13.5 (1.99)	14.2 (1.99)
**Sport Index**^**a**^ **at baseline (min/week)**					
Mean (SD)	438 (176)	130 (58.6)	407 (182)	157 (53.4)	281 (196)
**Difference Sport Index: T1 - T2 (min/week)**					
Mean (SD)	8 (230)	−44 (84)	−310 (190)	241 (198)	−71 (237)

^a^Sport Index (SI): total sum of leisure time sports activity within sport club, leisure time sports activity outside sport club, sports activity in age specific setting (school, kindergarten, work)

^b^high active: SI (T1) > 240 min/week & SI (T2) > 240 min/week

^c^low active: SI (T1)$\le$240 min/week & SI (T2)$\le$240 min/week

^d^decreaser: SI (T1) > 240 min/week & SI (T2)$\le$240 min/week

^e^increaser: SI (T1)$\le$240 min/week & SI (T2) > 240 min/week

The ANOVA revealed significant differences between the groups in CRF at the second measure (F(3,502) = 13.65, *p* < .001). The *post-hoc* analysis showed higher scores for the consistently high active group compared to the consistently low active group and the group that decreased their activity between the measures. Moreover, the consistently low active group had a significantly lower score than any other group at the second measurement (Table 2).

**Table 2:** Estimated Means of health outcomes at second measure by activity group

	High active^A^ *n* = 233	Low active^B^ *n* = 367	Decreasers^C^ *n* = 247	Increasers^D^ *n* = 100	Total *n* = 947
Endurance	**62.48**^**a,b**^ **(29.25)**	**43.17**^**a,c,d**^ **(28.28)**	**51.34**^**b,d**^ **(30.73)**	**59.35**^**c**^ **(29.16)**	52.09 (30.24)
PushUps	**59.65**^**e,f**^ **(27.01)**	**42.99**^**e**^ **(28.94)**	**49.27**^**f**^ **(27.81)**	51.05 (31.74)	49.99 (29.12)
Standing long jump	**59.86**^**g,h,i**^ **(27.36)**	**41.65**^**h,j**^ **(27.58)**	**50.92**^**i,j**^ **(27.89)**	**49.05**^**g**^ **(29.51)**	49.74 (28.65)
BMI	23.17 (3.28)	23.28 (4.86)	23.31 (4.49)	23.27 (3.75)	23.26 (4.26)

Endurance, Push-Ups & Standing long jump are reported in mean (SD) percentile score. BMI is calculated as mean (SD) weight in kilograms divided by height in meters squared. Bold indicates significant differences by post-hoc analyses between groups with the same superscript letters

Corresponding *p*-values for pairwise comparisons: ^a^: *p *< .001; ^b^: *p *= .004; ^c^: *p *< .001; ^d^: *p *= .034; ^e^: *p *< .001; ^f^: *p* = .004; ^g^: *p* = .020; ^h^: *p *< .001 ; ^i^: *p* = .022; ^j^: *p* = .009

^A^high active: SI (T1) > 240 min/week & SI (T2) > 240 min/week

^B^low active: SI (T1)$\le$240 min/week & SI (T2)$\le$240 min/week

^C^decreaser: SI (T1) > 240 min/week & SI (T2)$\le$240 min/week

^D^increaser: SI (T1)$\le$240 min/week & SI (T2) > 240 min/week

We found significant differences in Push-ups between the groups (F(3,599) = 10.45, *p* < .001). Tukey *post-hoc* tests indicate that the consistently high active group got significantly higher scores than the decreasers and the low active group. There were no significant differences between the decreasers, the increasers, and the low actives (Table 2).

The ANOVA for the standing long jump task showed significant differences between the group means (F(3,601) = 12.03, *p* < .001). The *post-hoc* analysis indicates that the consistently high active group achieved better scores than any other group and the “decreasers” performed better than the “low actives”.

The ANOVA for BMI showed no significant differences between the activity groups (F(3,610) = 0.08, *p* = .970).

The logistic regression for headache showed a significant overall effect for the activity groups (X^2^ = 17.2, df = 3, *p* < .001). The odds ratio (OR) (CI95%) of the consistently low active group was 2.1 (1.4, 3.2) compared to the consistently high active group for reporting recurrent headaches at the follow-up. The OR for the group that increased the PA was 2.5 (1.5, 4.3) and 1.9 (1.3, 3.0) for the group that decreased the PA respectively, compared to the consistently high active group.

## Discussion

The aim of this study was to examine whether maintaining, decreasing, or increasing activity levels over a five-year period during the transition from adolescence to adulthood has an effect on selected markers of physical health. CRF, MF, body composition, and recurrent pain were used as indicators of physical health. Overall, those who remained active or increased their sports activity had the most favorable health outcomes at follow-up.

CRF scores of the high actives were higher than those of the low actives and decreasers, but not higher than those of the increasers. Parts of this result are in contrast to those from other studies. Leppaenen et al. found that greater VPA and/or MVPA measured by the accelerometer at baseline were associated with better CRF one year later [34]. Buergi et al. report a favorable association between baseline PA and CRF in preschoolers [35], but it should be noted here that the population studied is considerably older. While this is adequate for the high actives compared to the low actives, the decreasers who had higher PA levels at baseline showed worse CRF scores at the follow-up compared to the actives. Whereas the CRF scores of the increasers, who had lower PA levels at baseline did not differ significantly from those of the active group. However, it should be noted that the interval between the two measurement points in the present study was approximately five years, whereas it was only one year in the study of Leppaenen. The positive correlation between higher PA and CRF does not seem to exist over the five-year period. The current PA level appears to be more important here, as the CRF of the active group in the study was not significantly better than the performance of those who increased their PA level between the measurements. In contrast, CRF levels were significantly higher in the increasers than in the low active, indicating that an increase in PA may lead to an improvement in endurance performance. A study by Kallio et al. found that even minor increases in PA resulted in a lower cardiometabolic risk later [36]. The study by Leppaenen et al. shows significant correlations with CRF only for MVPA and VPA. The study by Buergi et al. indicated that at least in the age group studied only baseline VPA is associated with an improvement in CRF [35]. This suggests that the intensity of PA plays a role in the effect. In the current study, no accelerometers were used; PA was assessed by questionnaire. The sports index used in the current study includes activities in sports clubs and sports in leisure time. It can be assumed that these activities tend to have a higher intensity than everyday activities, which is consistent with the results of the studies by Leppaenen and Buergi. At the same time, it should be noted that the participants in this study were considerably older.

From the area of MF, push-ups were used in this study as an indicator of upper limb strength, and standing long jump for lower limb strength. For the push-ups, it was found that the performance of the high-active participants was better than that of the decreasers and the low-active group, but not significantly higher than the performance of the increasers. The mean percentile score of the increasers was higher compared to the decreasers and the low active group, but the difference was not significant. For the standing long jump, the highly active group performed significantly better than any of the other groups. In addition, the decreasers were significantly better than the low active group. The results are consistent with the literature. Baquet et al. report that consistently high levels of PA are associated with better physical fitness [37]. A recent study of Swedish children aged four and nine years showed that the children who met the PA recommendations at both times had better fitness at the second measurement point [38].

For BMI, no differences were found between the activity groups in the current study. One possible factor here is the relatively long period of about five years between the surveys. During this time, other factors besides the PA may influence the development of the BMI. Another point is the fact that PA or physical training, e.g. to improve athletic performance, stimulates muscle growth and thus leads to an increase in body weight, which has a negative effect on the BMI value. To overcome this weakness of the BMI, other methods of assessing body composition are used that take into account whether the weight is due to body fat or lean mass. The aforementioned study by Leppaenen also found no effect of PA on BMI in the younger subjects. However, the authors found a weak negative correlation between PA and fat-free mass index (FFMI) or body fat percentage [34]. This is consistent with the results of a study by Metcalf et al. that examined the effect of achieving a government-recommended level of PA on obesity-related parameters. The study showed that children who were physically active above the recommended 3 METs achieved an improvement in their metabolic health, but no change in BMI or fatness [39]. In contrast, a systematic review concludes that the studies analyzed support the hypothesis that higher levels of habitual activity counteract the development of obesity [40]. However, the authors note that much of the evidence comes from cross-sectional studies and that longitudinal designs and intervention studies are needed to be able to make statements on causality [40].

Moreover, being consistently highly active was associated with less self-reported recurrent headaches at follow-up. The finding is consistent with previous cross-sectional analyses in different subsamples of the MoMo and KiGGS data [19, 30, 31].

In summary, the analysis showed that the consistently active group had a better CRF than the consistently less active group, a better MF than any other group, and was less likely to report recurrent headaches than the other groups. There was no difference in mean BMI between the activity groups.

Overall, our study is consistent with the evidence for beneficial longitudinal effects of PA on physical health. Participants who were consistently active in both surveys had better scores on health-related measures at the follow-up. The results underline the importance of an active lifestyle from early on, but they also suggest that becoming more active can improve physical health.

While the results of our study underline the importance of consistent PA for physical health, we know from other studies that PA declines during adolescence [41] and from adolescence to adulthood [42]. In light of this, appropriate promotion and the creation of conditions conducive to physical activity should ensure a high baseline level of physical activity in childhood and, at the same time, action should be taken to minimize the decline. Therefore, in addition to the question of possible factors influencing the decline, the development of appropriate programs and activities to promote PA must therefore also be the focus of future research.

Future research should look at whether there is a threshold value above which health effects become apparent and whether there may be a ceiling effect, i.e. a level above which no further improvement is achieved. Another interesting question is which types of sports or forms of organization have an effect and in what form.

### Strength and weaknesses

The analysis has some strengths and weaknesses that have to be considered. The study is based on a complex sample. The sampling strategy counteracts selection effects and allows representative statements to be made. In the case of the longitudinal sample, differences in re-participation rates between groups could lead to a skewed distribution and therefore reduce the generalizability of the results. However, the genuine longitudinal part of the study in which the same people are interviewed several times, offers the possibility of analyzing individual trajectories and improving the understanding of the development within a subject.

The interval between measurement times in the current study is relatively long with five years between the measurements. This means that an effect needs to be relatively stable and sufficiently clear in order to be recorded after this period. At the same time, the long time span offers a lot of room for possible confounding factors to influence the analyzed variables. One example of this is BMI, which is known to be significantly influenced by dietary behavior.

In the present analysis physical activity was assessed via questionnaire. Self-reported data tends to overestimate the amount of PA. This can be avoided or reduced by the use of sensors. On the other hand, the analysis deals with activity in specific settings, which in turn cannot be captured by sensors. Another point to consider is the type of PA being measured. In the case of the current study, an index representing the total amount of sports activity per week was used. Therefore, it can be assumed that most of the PA was structured, planned and, possibly guided. At the same time, the type of activity carried out was not taken into account.

## Conclusion

The results add to the evidence of the beneficial effects of PA on physical health [1, 2]. Thus, they support calls for an active lifestyle to promote and maintain health, such as those made by the WHO [43, 44].

The analysis suggests that consistent PA is the most beneficial option for maintaining and improving physical health. It follows that it is not only important to encourage people to be more physically active, but it is also important to support those who are active to stay active. Especially during the sensitive transitional phases in childhood and adolescence, such as entering secondary school or starting vocational training or university, it is important to have programs available that are adapted to the changing conditions.

Nevertheless, the results indicate that somebody with a lower level of PA can still improve their physical health by increasing their PA.

## Data Availability

Data and materials are available on resonable request from the corresponding author.
